# A Multimodal Knowledge-Based Deep Learning Approach for MGMT Promoter Methylation Identification

**DOI:** 10.3390/jimaging8120321

**Published:** 2022-12-03

**Authors:** Salvatore Capuozzo, Michela Gravina, Gianluca Gatta, Stefano Marrone, Carlo Sansone

**Affiliations:** 1 Department of Electrical Engineering and Information Technologies (DIETI), University of Naples Federico II, Via Claudio 21, 80125 Naples, Italy; 2 Department of Precision Medicine Division of Radiology, University of Campania Luigi Vanvitelli, 80138 Naples, Italy

**Keywords:** glioblastoma, convolutional neural network, MRI, MGMT promoter methylation

## Abstract

Glioblastoma Multiforme (GBM) is considered one of the most aggressive malignant tumors, characterized by a tremendously low survival rate. Despite alkylating chemotherapy being typically adopted to fight this tumor, it is known that O(6)-methylguanine-DNA methyltransferase (MGMT) enzyme repair abilities can antagonize the cytotoxic effects of alkylating agents, strongly limiting tumor cell destruction. However, it has been observed that MGMT promoter regions may be subject to methylation, a biological process preventing MGMT enzymes from removing the alkyl agents. As a consequence, the presence of the methylation process in GBM patients can be considered a predictive biomarker of response to therapy and a prognosis factor. Unfortunately, identifying signs of methylation is a non-trivial matter, often requiring expensive, time-consuming, and invasive procedures. In this work, we propose to face MGMT promoter methylation identification analyzing Magnetic Resonance Imaging (MRI) data using a Deep Learning (DL) based approach. In particular, we propose a Convolutional Neural Network (CNN) operating on suspicious regions on the FLAIR series, pre-selected through an unsupervised Knowledge-Based filter leveraging both FLAIR and T1-weighted series. The experiments, run on two different publicly available datasets, show that the proposed approach can obtain results comparable to (and in some cases better than) the considered competitor approach while consisting of less than 0.29% of its parameters. Finally, we perform an eXplainable AI (XAI) analysis to take a little step further toward the clinical usability of a DL-based approach for MGMT promoter detection in brain MRI.

## 1. Introduction

Glioblastoma Multiforme (GBM) is considered one of the most aggressive malignant tumors beginning within the brain, cerebellum, and brain stem [1]. Despite being a rare tumor when compared with the incidence of all other malignancies, it is the most common primary brain tumor in adults, often diagnosed in patients between 45 and 70 years old. It is considered one of the most threatening dangers to humans due to its typical unfavorable prognoses, low survival rate, and rapidly progressive course. Moreover, its nonspecific symptoms and unknowable causes (except when arising after therapeutic irradiation to the brain performed for another disease) bring uncertainty and dejection to sick patients, making GBM prevention extremely hard and often late diagnosed. In clinical trials, the diagnostic procedures consist of biopsies and neurological exams, invasive and time-consuming practices that further complicate rapid and effective diagnosis. To cope with this, Magnetic Resonance Imaging (MRI) is more and more preferred as a non-invasive diagnostic tool for GBM early detection [2].

Once diagnosticated, neurosurgery, radiation therapy, and chemotherapy are the possible treatments [3]. In more detail, chemotherapy based on alkylating agents is used since it attaches alkyl groups to tumor cells’ DNA to damage it and prevents cell replication. Despite chemotherapy, it is known that O(6)-methylguanine-DNA methyltransferase (MGMT) enzyme repair abilities can antagonize the cytotoxic effects of alkylating agents, strongly limiting tumor cell destruction. However, it has also been observed that MGMT promoter regions may be subject to methylation, a biological process preventing MGMT enzymes from removing the alkyl agents [4]. As a consequence, the presence of the methylation process in GBM patients can be considered as a predictive biomarker of response to alkylating therapies and thus a favorable prognosis factor [5]. Therefore, the quick and effective identification of methylation activation is becoming an urgent matter for effective GBM therapy assessment. Unfortunately, identifying signs of methylation is a non-trivial matter, often requiring expensive, time-consuming, and invasive procedures. Indeed, although studies on methylation detection by means of Machine Learning (ML) approaches on MRI scans are promising [6,7], they still do not report an absolute correlation between radiomics features and the MGMT promoter methylation [8,9].

To support this line of research, recently the Radiological Society of North America (RSNA) and the Medical Image Computing and Computer Assisted Intervention Society (the MICCAI Society) have jointly launched a competition (https://www.kaggle.com/competitions/rsna-miccai-brain-tumor-radiogenomic-classification, accessed on 13 July 2021) to identify the genetic subtype of glioblastoma using MRI with the aim of detecting the presence of MGMT promoter methylation. Despite being far from conclusive, results achieved by different teams seem to suggest that some correlations may actually exist and can be found by using Deep Learning (DL) approaches. Nonetheless, several participants highlighted the difficulties associated with (i) the high inter-subject variability and (ii) the resulting need for a wider amount of data to train huge DL models.

To cope with these problems, in this work, we introduce a new simple but effective DL-based approach able to perform better than the official competition winner while consisting of less than 0.29% of its parameters (*14,356,929* of the winner versus *40,561* of the proposed approach). More in detail, we propose a Convolutional Neural Network (CNN), a particular artificial neural network consisting, among others, of convolutional layers able to autonomously learn a set of morphological and textural features that fit the specific task to solve. Moreover, leveraging the fact that *medical images are more than pictures* [10], in the proposed methodology we have also implemented a *multimodal Knowledge-Based Filtering (KBF)* approach to serve as an early fusion technique to merge information coming from two different MRI series. In particular, we fuse the T1-weighted (T1-w) and the Fluid Attenuated Inversion Recovery (FLAIR) series, both very common in brain MRI, with the aim of retrieving as much useful information as possible from patients. The resulting system consists of a supervised approach operating on suspicious regions on the FLAIR series, pre-selected through an unsupervised knowledge-based filter leveraging both FLAIR and T1-weighted series. To estimate the effectiveness of the proposed approach in a real clinical context we also tested our approach on the UPENN-GBM dataset (https://wiki.cancerimagingarchive.net/pages/viewpage.action?pageId=70225642, accessed on 15 June 2022) [11]. Finally, to try to limit the impact of the highlighted inter-patient variability, all the experiments have been executed by using a 5-fold cross-validation approach.

The rest of the paper is organized as follows: Section 2 briefly analyzes the current literature, with an emphasis on the limits of current proposals; Section 3 introduces the considered datasets; Section 4 describes the implemented methodology; Section 5 illustrates the experimental setup; Section 6 reports the obtained results; finally Section 7 provides some conclusions.

## 2. Related Works

In recent years, a few approaches were explored to build an efficient MGMT promoter methylation detector. Most of them adopt DL techniques, in particular CNNs, which are used to detect distinctive methylation features in the tumor areas, both in 2D slices and in 3D brain volumes. In 2018, L. Han et al. [12] proposed a hybrid solution exploiting CNNs and bidirectional recurrent neural networks, called CRNN, as an alternative to the standard 3D CNN. The bidirectional RNN allows the gathering of patient slices providing a unique methylation state. The considered datasets come from The Cancer Imaging Archive, including T1-w, T2-w and FLAIR sequences, and from The Cancer Genome Atlas for methylation sites. The implemented solution obtains accuracy and AUC scores on the test set of 62% and 61%, respectively. In 2021, Yogananda et al. [13] proposed a powerful model for MGMT promoter methylation detection that achieved nearly 95% on the test set. Its effectiveness was possible due to the available data since both T2-w sequences and tumor segmentation masks were provided. In 2022, S. Chen et al. [14] exposed a complete solution for methylation detection, giving the opportunity to work both in single and in multimodality exploiting T1-w, T2-w, Apparent Diffusion Coefficient (ADC) and Contrast-Enhanced (CE) T1-w MRI sequences. This solution is based on the ResNet model [15] and relies on manually segmented slices in order to focus only on tumor areas. In the same year, another solution was proposed by S. Das [16], using datasets provided by the BraTS 2020 and 2021 challenges, that included both MRI slices and tumor masks. The authors built an adversarial architecture based on an enhanced ResNet model gaining accuracy and AUC scores on the test set of about 66%. In particular, the authors focused on the BraTS 2020 and 2021 datasets to train a model for tumor segmentation, while the dataset presented in the Brain Tumor AI Challenge (https://www.kaggle.com/competitions/rsna-miccai-brain-tumor-radiogenomic-classification) [17] is used for MGMT promoter methylation detection. Moreover, the solution proposed in [16] strongly highlights the need for a mask that gives information about tumor localization.

It is worth noting that despite the reported solutions exploiting different models and approaches, they all share the need for detailed information about methylation sites or segmented tumor areas. However, this is rarely available in a real scenario, to the point that even the Brain Tumor AI Challenge (https://www.kaggle.com/competitions/rsna-miccai-brain-tumor-radiogenomic-classification) [17] highlighted the need for approaches able to work directly on MRI data, without further information about tumor location or segmentation. Taking this characteristic into account, a recent work [18] tried to understand if the methylation detection task was possible with DL approaches without segmentation masks, using the data from the Brain Tumor AI Challenge (https://www.kaggle.com/competitions/rsna-miccai-brain-tumor-radiogenomic-classification) [17]. After proposing their model based on a 3D variant of the EfficientNet [19], they had to admit that, even if a model can be trained with methylation labels exploiting MRI sequences as T1-w, T2-w, CE T1-w and FLAIR, it cannot have great performance, showing an average AUC on the test set of 58%. Along the same lines, the winner of the competition, the Tunisia.ai team, reached only 62% of AUC on the test set fine-tuning a massive residual network consisting of more than 14 M parameters. These results, together with the performance shown on the challenge leaderboard and with the high inter-patient variability highlighted by the participants during the competition, suggest that it is important to implement a (possibly unsupervised) way to automatically obtain the area of the tumor and, in turn, design a smaller architecture able to better generalize despite the reduced number of available samples for each genetic expression of the methylation.

## 3. Considered Cohorts

As for most biomedical tasks, identifying a suited sample of subjects properly representing the real population is a non-trivial task. To try to limit the impact of this choice and to estimate the clinical effectiveness of the proposed approach, in this paper, we focus on two datasets:

- The first one is provided by the Brain Tumor AI Challenge (https://www.kaggle.com/competitions/rsna-miccai-brain-tumor-radiogenomic-classification) [17], consisting of 573 subjects obtained by merging the training and validation sets available in the competition. This dataset is composed of 303 patients with MGMT promoter methylation and 270 without. The dataset uses DICOM files, that include a list of metadata in the form of a set of tags, such as Image Orientation, Slice Location, Pixel Spacing, and Spacing Between Slices that are used to generate the acquisition volumes.
- The second dataset is the UPENN-GBM one (https://wiki.cancerimagingarchive.net/pages/viewpage.action?pageId=70225642) [11], consisting of 291 subjects for whom the information about the MGMT promoter methylation is available, of which 121 with methylation and 170 without. Similarly to the first dataset, the UPENN-GBM [11] uses the DICOM file format. This dataset comes from scans obtained from GBM patients of the University of Pennsylvania Health System, which contain other clinical information such as overall survival and patients’ demographics.

Both datasets include Fluid Attenuated Inversion Recovery (FLAIR), T1-weighted (T1w), T1-weighted post-contrast (T1wCE) and T2-weighted (T2w) MRI sequences.

## 4. Proposed Approach

**Figure 1:**
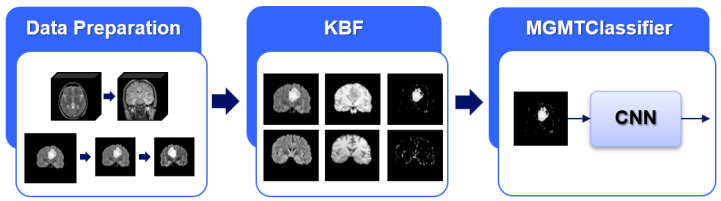
Exemplified schema of the proposed approach: on the left, the *Data Preparation* step generates isotropic and normalized acquisitions; in the middle, the *Knowledge-Based Filtering (KBF)* step leverages the medical knowledge to pre-select, in an unsupervised manner, the ROI corresponding to suspect lesions; on the right, the *MGMT promoter methylation identification* step adopts a CNN for the identification of the methylation process.

In this paper, we propose a DL-based approach for MGMT promoter methylation identification leveraging medical knowledge to deal with the lack of tumor segmentation masks. In more detail, the implemented solution consists of three main blocks, as summarized in Figure 1: the *Data Preparation* step, generating isotropic and normalized acquisitions; the *Knowledge-Based Filtering (KBF)*, leveraging the medical knowledge to pre-select, in an unsupervised manner, the Region of Interest (ROI) corresponding to possibly tumor regions in the MRI scans; the *MGMT promoter methylation identification*, using a 2D or 3D CNN for the identification of a methylation process. The next sections detail each module, highlighting input and output while explaining the rationale behind the choices made.

### 4.1. Data Preparation

**Figure 2:**
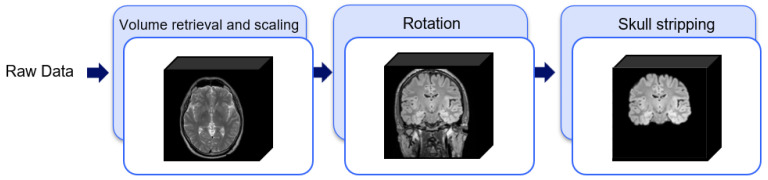
Illustration of the processes involved within the “Data Preparation” step.The volume retrieval and scaling create the 3D acquisitions with the isotropic dimension of 1×1×1 mm${}^{3}$; the Rotation step creates a set of volumes in the sagittal projection; the Skull stripping removes the tissue outside the brain.

In MRI acquisition, the slices are stacked into 3D volumes representing the brain. As reported in [11,17], the datasets used in this paper were heterogeneously obtained from different scanners and acquisition protocols from multiple institutions, resulting in the need for implementing several steps to prepare volumes, before using them in the proposed methodology. In both datasets, the *Data-Preparation* step consists of volume retrieval, co-registration of acquisitions to the same anatomical template [20], inter-modality registration, scaling and rotation to have acquisitions with the same isotropic dimension of 1 mm and spatial orientation. In particular, the volume retrieval focuses on the creation of the 3D volumes representing the MRI scans, considering the DICOM files. All the slices are ordered using the *Slice Location* tag, available in each DICOM file, obtaining for each patient a set of aligned acquisitions for the co-registration as proposed in [20] and the inter-modality registration step using a rigid transformation. Each voxel in the generated volume is linked to information about its millimeter measurement (mm). The property *Pixel Spacing*, which is determined by two values $\left(\hat{x^{p}},\hat{y^{p}}\right)$ that represent the row and vertical spacing, specifies the physical separation between the centers of each two-dimensional pixel during the acquisition of the MRI sequence for the patient *p*. Additionally, the *Spacing Between Slices* feature, denoted by the numeric value $\hat{z^{p}}$, describes the separation between slices as determined along the first image’s normal. This means that each voxel represents a volume with dimensions of $\hat{x^{p}}\times \hat{y^{p}}\times \hat{z^{p}}$ mm${}^{3}$, which is the resolution of the MRI image for the patient *p*. Since various subjects’ resolutions might differ, all patient volumes are equally scaled to provide acquisitions with an isotropic size of 1×1×1 mm ${}^{3}$. Moreover, the *Image Orientation* attribute specifies the direction cosines of the first row and the first column with respect to the patient, and it is composed of three two-element vectors for the x,y and *z* axes directions. The information included in the above tag enables a proper rotation of the isotropic volume to a standard patient orientation space. At the end of the data preparation module, all the volumes will have a sagittal orientation. Since volumes may include extra-cerebral tissues, which are not required for our purposes, a skull stripping process is performed. This process requires the adoption of a 3D semantic segmentation network for brain detection in order to generate a brain mask, which will be used to crop what is outside of it. In this case, we exploit the HD-BET tool [21], based on a 3D U-Net. It is worth noting that the skull stripping procedure is applied on UPENN-GBM one (https://wiki.cancerimagingarchive.net/pages/viewpage.action?pageId=70225642) [11], since the pre-processing implemented by the authors on the dataset provided in the Brain Tumor AI Challenge (https://www.kaggle.com/competitions/rsna-miccai-brain-tumor-radiogenomic-classification) [17] included this step. The pipeline of *Data-Preparation* step is summarised in Figure 2.

### 4.2. Knowledge-Based Filtering (KBF)

**Figure 3:**
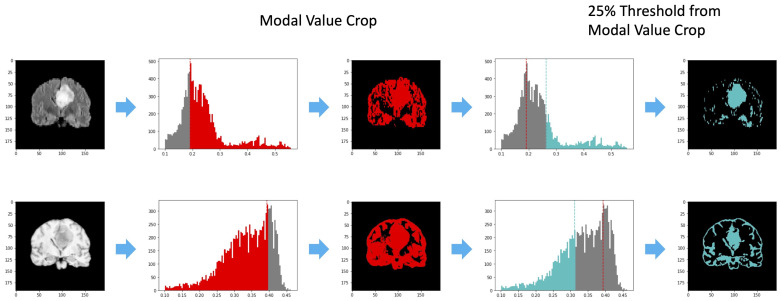
An illustrative example of the threshold operations performed during the KBF procedure on FLAIR and T1w slices (first image), reported in the first and second rows respectively. The red filter (second image) considers the mode value, while the third image represents the output of the first filtering process. On the other hand, the light-blue filter (fourth image) exploits the 25% of the highest and the 25% of the lowest values in the FLAIR and T1-w acquisition, respectively. The output of the KBF procedure is shown in the fifth image.

**Figure 4:**
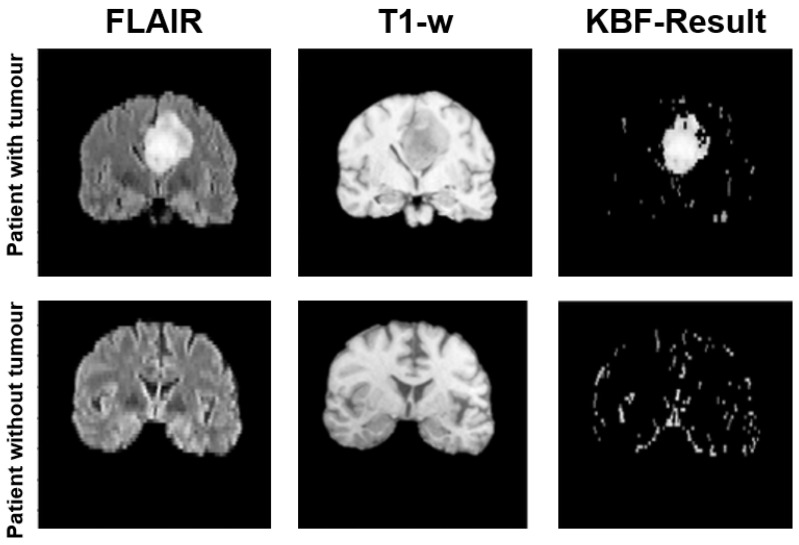
An illustrative example of the results produced by the KBF module on two patients: if the slice contains a tumor (**top row**) a huge cluster is generated in the preselection mask, while in the opposite case (**bottom row**) the mask contains sparse outliers.

As described in Section 2, determining the ROI representing the tumor region when the segmentation mask is not available is a crucial step. In this work, we propose to select the area of interest in an unsupervised manner, leveraging past medical experience as proposed in [22,23] for tumor recognition. In particular, we exploit two very simple characteristics of lesioned tissues in the considered series: in T1-W slices, tumor areas have pixels whose intensity is higher than cerebrospinal fluids but lower than any other kind of tissue; in FLAIR slices, pixels with the highest intensity belong to the tumor region. Leveraging these characteristics, from each input volume it is possible to preselect potentially lesioned tissues by applying a threshold on the histogram of the signal intensities occurrences. Since tumor areas are characterized by pixels with high intensity in the FLAIR and low values in the T1-W, the most common value in terms of signal intensity (mode) can be used to split the available information and remove one of the two generated subareas. In particular, pixels with an intensity higher than the mode are considered in the FLAIR acquisition, while pixels with an intensity lower than the mode are retained in the T1-w volume. Then, the remaining pixels are sorted by the intensity value and undergo a further threshold operation, which considers the 25% of the highest and the 25% of the lowest values in the FLAIR and T1-w acquisition, respectively. Figure 3 provides an illustrative example of the threshold operations implemented in Knowledge-Based Filtering (KBF). Non-significant values in terms of intensity near the intensity modal value are removed, as they are very unlikely to represent tumor areas. Since tumor pixels have high intensity on FLAIR and low intensity on T1-w, a cross-intersection between the highest values of the first and lowest values of the second acquisition is required. Despite this possibly causing the loss of some pixels for the tumor due to their similarity with those of cerebrospinal fluids in T1-W, this is not a real concern in our case, as the main aim of the procedure is to localize the tumor and not to perform a pixel-level segmentation. The output of this process is a mask consisting of huge clusters corresponding to the ROIs (i.e., possibly lesioned areas) and little outliers if the slice has a tumor or sparse outliers in the opposite case. Figure 4 shows an example of KBF application on two MRI acquisitions belonging to patients with and without cancer, respectively. The resulting ROI is used to select from the FLAIR sequence the portion of the image to be considered by the actual methylation detection module, implementing a point-wise multiplication. We chose the FLAIR sequence for the good performance shown in the literature for tasks related to lesion diagnosis [24,25].

**Figure 5:**
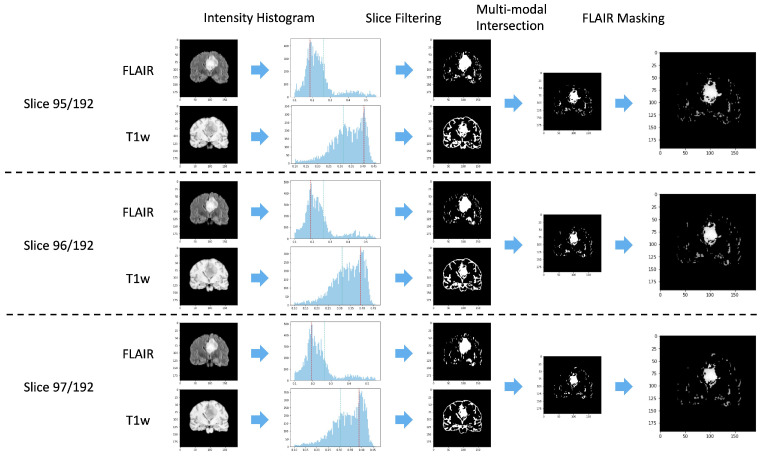
An illustrative example of how the KBF module operates on three consecutive couples of FLAIR and T1-W slices: the threshold based on the mode value is represented in red, while the light-blue line represents the threshold at 25% of the values for the FLAIR and T1-W acquisitions.

The proposed KBF module aims to compensate for the lack of segmentation masks, reducing the effort required by the physicians and making our methodology applicable to datasets where tumor areas are not identified. The KBF exploits properties of T1-w and FLAIR sequences, resulting in a multi-modal knowledge-based pre-processing procedure. In particular, we implement an early fusion technique, in which information coming from multiple sources is merged to highlight different characteristics [26]. In this paper, the two sequences are exploited to create a mask representing the area to consider. Figure 5 summarizes the KBF procedure, showing the results on three central slices.

To reduce the amount of data to process, we crop each FLAIR volume considering the smallest cubical box around the brain, obtaining acquisitions of size 192×192×192. It is worth noting that the choice of the box is computed considering the characteristics of both datasets, and ensuring that pixels belonging to the area identified by the KBF are not removed. Moreover, the obtained volume is normalized in [0, 1] on a patient basis to ensure that, in the next stage, the considered CNNs operate on images having the same scale across different acquisitions. Furthermore, it is worth noting that, since the KBF procedure aims to select the tumor area inside the brain, it is robust against an incomplete removal of the skull, which may occur in the Data Preparation step.

### 4.3. MGMT Promoter Methylation Identification

**Figure 6:**
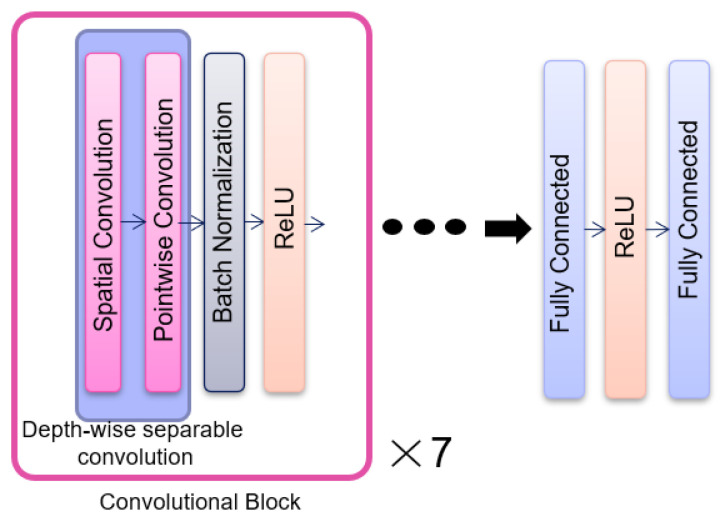
The MGMTClassifier architecture consisting of seven convolutional blocks with depth-wise separable convolutions spaced by batch normalization and ReLU as activation function, followed by two fully connected layers and a ReLU activation.

We exploit CNN to face the task of MGMT promoter methylation identification. In particular, we introduce the *MGMTClassifier*, a sequential network with seven convolutional blocks and two fully connected layers separated by the Rectified Linear Unit (ReLU) as an activation function, whose architecture is represented in Figure 6. More in detail, each convolutional block consists of a convolutional layer, followed by batch normalization and ReLU function, responsible for the dimensionality reduction in the input feature map while doubling the number of the channels, except for the first convolutional block with eight output channels. To reduce the number of training parameters while avoiding overfitting, we adopt depth-wise separable convolution [27] in each convolutional layer that consists of implementing two operations: the former acts in the spatial dimension (spatial convolution) without changing the number of channels, while the latter is a pointwise convolution that determines the output channels. This architecture has been designed taking into consideration the morphological characteristics of the brain in DCE-MRI. Indeed, the proposed structure works with both 2D and 3D convolutional layers, allowing the use of the preferred version based on the characteristic of the dataset (e.g., spacing between slices, number of available samples, etc.). In both cases, the convolutional layers in the proposed architecture consist of operations where the spatial convolution uses 3×3 (×3) kernel, with a stride and padding set to 2 and 1, respectively, while the pointwise convolution presents a 1×1 (×1) kernel with a stride set to 1 and without padding. Finally, to improve the network robustness by introducing variability in the set of data used for training, we use classical data augmentation techniques, such as random rotations and flipping.

## 5. Experimental Setup

As described in Section 3, we perform experiments on two different datasets: one provided by the Brain Tumor AI Challenge (denoted as dataset “*A*” hereafter) and another gathered by the University of Pennsylvania (denoted as dataset “*B*” hereafter). We tested the proposed methodology on both datasets separately, also performing experiments by merging them to further assess the generalization ability of the designed approach. All the experiments were executed using a 5-fold cross-validation strategy. It is worth noting that we did not consider the test set provided by the Brain Tumor AI Challenge (https://www.kaggle.com/competitions/rsna-miccai-brain-tumor-radiogenomic-classification) [17] since the labels were (and are, at the time of writing this paper) not available. Moreover, as highlighted by several participants, the high inter-intra patient variability has resulted in huge variations between public and private leaderboards. We merged the public train and validation set, before defining the folds to perform the experiments. Using a CV strategy allows for the reduction in the variations associated with a fortunate/unfortunate split, thus increasing the reliability of the results.

As described in Section 4.3, the proposed CNN can be implemented using both 2D and 3D convolutional layers. For the sake of completeness, we experimented with both, trying to highlight the pros and cons of both solutions. During the experiments, the maximum number of epochs has been set to 150, the batch size to 8 to $5\times 10^{-4}$, using the Adam optimizer. Performance was evaluated in terms of Accuracy (ACC), Specificity (SPE), Sensitivity (SEN), Precision (PRE), and Area under the ROC Curve (AUC). In particular, ACC represents the percentage of corrected classified instances, while SEN and SPE are used in the binary classification task to assess the true positive and true negative rates, respectively. In this paper, we consider as *positive* volumes in which the methylation process is present and as *negative* the others. As a consequence, SEN corresponds to the fraction of methylation cases correctly identified, whilst SPE acts on the MRI volumes in which this process is not available (negative cases), reporting the portion of them properly predicted by the implemented model. As aforementioned in Section 1, the presence of the methylation process is a favorable prognosis factor since it prevents the MGMT enzymes from removing the alkylating agents. This characteristic suggests that an error in the negative samples leads to an underestimation of tumor severity as a prognosis more favorable than the actual one predicted. Finally, the AUC is an essential performance measurement since it evaluates the ability of the model to distinguish between two classes.

To better frame the results achieved by the proposed approach, we also compared (under the same 5-cv experimental setup) it against the solution proposed by Tunisia.ai, the competition-winning team, proposing to implement a 3D residual network trained from scratch considering only the T1-w CE sequence. For the comparison, we use the code that the team released on the competition website. As aforementioned, in this paper, we did not use the test set provided by the competition, where the winners achieved 62% of AUC since labels have not been made public. The use of a 5-fold cv proved a more robust evaluation than the hold-out implemented in the competition. Our aim is to compare two different approaches that are the one presented in this paper and the solution proposed by Tunisia.ai, in which we retain the input sequence used by the team (T1-w CE). All the experiments were run using Python 3.9, with the proposed CNN implemented in PyTorch (version 1.10). We used a Linux workstation equipped with AMD Ryzen 7 5000 (AMD, Sunnyvale, CA, USA) and an 8 GB DDR4 RAM NVIDIA RTX 3080 (NVIDIA, Santa Clara, California). All the codes used to derive the results reported in this paper will be made available to the research community (The code is available here: https://github.com/priamus-lab/GBM-MGMT-Detection, accessed on 18 September 2022).

## 6. Results

In this section, we report the results obtained by the proposed approach on dataset *A*, *B* and on their union, in terms of the performance metrics described in Section 5 under the described 5-fold cross-validation scenario. Table 1 shows the results obtained considering the dataset *A*. The first two rows report the performance achieved with the 3D MGMTClassifier and 2D MGMTClassifier, respectively, while the last one shows the comparison with the solution proposed by the team Tunisia.ai. It is possible to note that the configuration based on the 2D MGMTClassifier has the highest performance in terms of ACC (57.77%), SPE (54.44%), PRE (59.93%), and F1 (63.33%). Similarly, Table 2 reports the performance of the implemented experiments on the *B* dataset. In this case, the 3D MGMTClassifier outperforms the other models by a wide margin, achieving 60.06% in ACC, 74.03% in SPE, 64.40% in PRE and 52.53% in F1.

As aforementioned, to further assess the generalization ability of the proposed approach, we also experimented with a *cross-dataset* scenario. In particular, Table 3 reports the results of the models trained on dataset *A* and tested on *B*, while Table 4 shows the performance of the networks trained on dataset *B* and tested on *A*. In both cases, we retain the same 5-fold CV division, making the results in Table 3 and Table 4 comparable with those presented in Table 2 and Table 1, respectively. Despite the fact that in both cases there is a reduction in performance, it is interesting to note that our approach tends to perform in a more robust manner, showing an overall behavior coherent with the one had in the single dataset scenarios. Indeed, Tunisia.ai model presents a huge gap in performance when the network trained on A is tested on B, obtaining 37.30%, 26.72%, 36.54%, and 49.58% in ACC, SPE, PRE, and AUC. For the sake of completeness, in Table 5, we report the results obtained by merging both datasets into a *A+B* setting. In this case, we still implement a 5-fold CV by merging, in each iteration, the corresponding folds previously identified on datasets A and B separately.

## 7. Discussion and Conclusions

In this work, we introduced a new approach leveraging deep learning and unsupervised voxel pre-selection to perform MGMT promoter methylation identification in brain MRI when suspect lesion masks are not available. In particular, we propose a Convolutional Neural Network (CNN) operating on suspicious regions on the FLAIR series (using 2D or 3D convolutional filters, based on the amount of available data), pre-selected through an unsupervised Knowledge-Based filter leveraging both FLAIR and T1-weighted series. To estimate the effectiveness of the proposed approach, we performed experiments on two different datasets: the Brain Tumor AI Challenge (https://www.kaggle.com/competitions/rsna-miccai-brain-tumor-radiogenomic-classification) [17], a competition that started in July 2021, with more than 1500 teams taking part; the UPENN-GBM one (https://wiki.cancerimagingarchive.net/pages/viewpage.action?pageId=70225642) [11], consisting of subjects from the University of Pennsylvania Health System. For both datasets, we compared our approach (both 2D and 3D versions) against the official Brain Tumor AI Challenge winner, under a 5-fold cross-validation strategy to reduce the high inter-intra patient viability.

When the two datasets are considered separately (Table 1 and Table 2), results show that the proposed approach performs, in some cases sensibly, better than the considered competitor. Interestingly, the cross and mixed dataset scenarios are less consistent, with the proposed approach and the competitor outperforming each other on different metrics. This further confirms the high variability associated with MGMT promoter detection. Focusing on the structures of the considered approaches (both the proposed ones and the competitor), it is worth noting that they are quite different in terms of size and required training time. Indeed, while the Tunisia.ai approach is based on a ResNet [15] model consisting of more than 14 M trainable parameters, the proposed approach only consists of 183,000 and 22,000 parameters, respectively, for the 3D and 2D versions. This, turn, results in a training time of ∼30 s per epoch for the proposed approaches versus ∼5 m per epoch for the Tunisia.ai model, considering in both cases the same hardware (Section 5). This suggests that considering a bigger dataset, the proposed approach could potentially perform even better.

**Figure 7:**
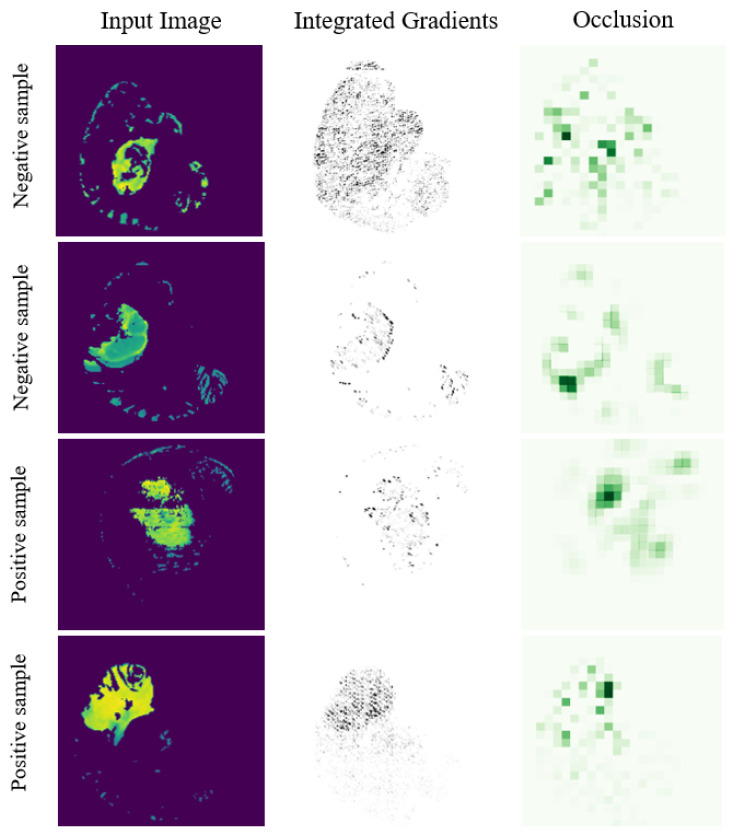
Results of the Integrated Gradients [28] and Occlusion [29] models considering four different inputs volumes. In the first two rows, we consider negative samples without the methylation process. The last two rows show positive instances, in which the methylation is present.

One of the biggest concerns associated with using AI models in a real clinical context, especially when performances are not astonishing, is associated with their trustworthiness. Thus, we also report some Explainable-AI (XAI) analyses to assess the interpretability of the solution showing the best performance (i.e., the 3D MGMTClassifier). In particular, we use the Integrated Gradients [28] and Occlusion [29] approaches from Captum [30], an open source library built on PyTorch. In particular, the former is an interpretability algorithm that assigns an importance score to each input feature, while the latter consists in a perturbation-based approach that computes the importance of each region by evaluating the differences in the output when the selected area is replaced (occluded) with a given baseline (i.e., zero value). The result of the Occlusion [29] method is a mask in which the most critical areas show an intense value. Figure 7 shows the results of the Integrated Gradients [28] and Occlusion [29] models considering four different input volumes. In the first two rows, we consider negative samples in which the methylation process is absent, while in the last rows, we report positive instances, in which the methylation is present. It is worth noting that the images shown in Figure 7 are correctly classified by the implemented model and, even if the input is a 3D volume, we report only the slice with the highest information content for clarity in the visualization. As we expected, the results of the Integrated Gradients [28] model suggest that only the pixels within the ROI identified by the KBF are considered, thus exploiting the tumor area. Moreover, the Occlusion method [29] considers the tumor region as a critic only in the case with methylation, as reported in the third and fourth rows, while in patients without methylation it seems that the area surrounding the tumor strongly affects the output, making the network change its prediction if that part is occluded. This further support the idea that, when an ROI is not available, even a simple unsupervised pre-selection stage can support the reliability of a DL-based approach.

Besides the reported results, when evaluating the solutions implemented only by considering the competition dataset (https://www.kaggle.com/competitions/rsna-miccai-brain-tumor-radiogenomic-classification), it is possible to note a big difference in terms of performance between the solutions proposed in the literature (i.e., 95% of accuracy) and those proposed in this work, as well as those submitted to the competition (as visible from the competition leaderboard and from a recap work reported in [18]). We strongly argue that, despite our approach partially coping with this, the absence of tumor segmentation masks poses severe limits to the performance that a DL-based approach can achieve for the MGMT promoter detection task. After finding out that the KBF pipeline makes the solution better than the ones participating in the Brain Tumor AI Challenge, the focus for future works will be on improving the filtering, in order to remove small clusters, and on building new, more sophisticated, and tailor-made neural networks, so that it can better identify methylation features, and to test an intermediate multimodal fusion configuration (e.g., a Y-shaped network).

## Figures and Tables

**Table 1:** 5-fold CV performances of models trained and tested on dataset *A*. The input sequence is KBF for both the 3D and 2D MGMTClassifier models and T1-w CE for the Tunisia.ai one. For each metric, the best value is reported in bold.

Model	ACC	SPE	SEN	PRE	F1	AUC
3D MGMTClassifier	55.09%	50.34%	59.74%	55.18%	57.37%	**55.38%**
2D MGMTClassifier	**57.77%**	**54.44%**	60.73%	**59.93%**	**60.33%**	53.55%
Tunisia.ai	52.31%	33.45%	**69.38%**	53.52%	60.30%	53.84%

**Table 2:** 5- fold CV performances of models trained and tested on dataset *B*. The input sequence is KBF for both the 3D and 2D MGMTClassifier models and T1-w CE for the Tunisia.ai one. For each metric, the best value is reported in bold.

Model	ACC	SPE	SEN	PRE	F1	AUC
3D MGMTClassifier	**60.06%**	**74.03%**	45.35%	**62.40%**	**52.53%**	**59.80%**
2D MGMTClassifier	55.66%	62.98%	45.31%	46.40%	45.85%	55.57%
Tunisia.ai	55.14%	54.31%	**56.52%**	42.39%	48.45%	57.56%

**Table 3:** 5-fold CV performances of models trained on dataset *A* and tested on dataset *B*. The input sequence is KBF for both the 3D and 2D MGMTClassifier models and T1-w CE for the Tunisia.ai one. For each metric, the best value is reported in bold.

Model	ACC	SPE	SEN	PRE	F1	AUC
3D MGMTClassifier	48.99%	57.80%	40.16%	**48.68%**	**44.01%**	48.78%
2D MGMTClassifier	**52.58%**	**59.41%**	42.98%	42.98%	42.98%	**51.51%**
Tunisia.ai	37.30%	26.72%	**55.07%**	36.54%	43.93%	49.58%

**Table 4:** 5-fold CV performances of models trained on dataset *B* and tested on dataset *A*. The input sequence is KBF for both the 3D and 2D MGMTClassifier models and T1-w CE for the Tunisia.ai one.For each metric, the best value is reported in bold.

Model	ACC	SPE	SEN	PRE	F1	AUC
3D MGMTClassifier	49.47%	**65.94%**	33.00%	49.21%	39.51%	50.57%
2D MGMTClassifier	51.66%	51.85%	51.49%	**54.55%**	52.98%	50.72%
Tunisia.ai	**51.93%**	28.35%	**73.29%**	52.90%	**61.45%**	**50.83%**

**Table 5:** 5-fold CV performances of models trained and tested on dataset *A*+*B*. The input sequence is KBF for both the 3D and 2D MGMTClassifier models and T1-w CE for the Tunisia.ai one. For each metric, the best value is reported in bold.

Model	ACC	SPE	SEN	PRE	F1	AUC
3D MGMTClassifier	56.81%	**65.13%**	48.58%	**58.44%**	53.06%	57.59%
2D MGMTClassifier	53.74%	48.11%	59.63 %	52.34%	55.75%	55.17%
Tunisia.ai	**56.88%**	48.22%	**65.96%**	54.87%	**59.91%**	**58.63%**

## Data Availability

The data used in this paper is part of the publicly available Brain Tumor AI Challenge and the UPENN-GBM datasets.

## References

[1] Hanif F., Muzaffar K., Perveen K., Malhi S.M., Simjee S. (2017). Simjee Glioblastoma Multiforme: A Review of its Epidemiology and Pathogenesis through Clinical Presentation and Treatment. Asian Pac. J. Cancer Prev.

[2] Shukla G., Alexander G.S., Bakas S., Nikam R., Talekar K., Palmer J.D., Shi W. (2017). Advanced magnetic resonance imaging in glioblastoma: A review. Chin. Clin. Oncol..

[3] Young R.M., Jamshidi A., Davis G., Sherman J.H. (2015). Current trends in the surgical management and treatment of adult glioblastoma. Ann. Transl. Med..

[4] Christmann M., Verbeek B., Roos W.P., Kaina B. (2011). O6-Methylguanine-DNA methyltransferase (MGMT) in normal tissues and tumors: Enzyme activity, promoter methylation and immunohistochemistry. Biochim. Biophys. Acta (BBA)-Rev. Cancer.

[5] Haque W., Thong E., Andrabi S., Verma V., Butler E.B., Teh B.S. (2021). Prognostic and predictive impact of MGMT promoter methylation in grade 3 gliomas. J. Clin. Neurosci..

[6] Ahn S.S., Cha S. (2021). Pre-and Post-Treatment Imaging of Primary Central Nervous System Tumors in the Molecular and Genetic Era. Korean J. Radiol..

[7] Suh C., Kim H., Jung S., Choi C., Kim S. (2018). Clinically relevant imaging features for MGMT promoter methylation in multiple glioblastoma studies: A systematic review and meta-analysis. Am. J. Neuroradiol..

[8] Han Y., Yan L.F., Wang X.B., Sun Y.Z., Zhang X., Liu Z.C., Nan H.Y., Hu Y.C., Yang Y., Zhang J. (2018). Structural and advanced imaging in predicting MGMT promoter methylation of primary glioblastoma: A region of interest based analysis. BMC Cancer.

[9] Ding Y., Yang Q., Wang B., Ye G., Tong X. (2016). The correlation of MGMT promoter methylation and clinicopathological features in gastric cancer: A systematic review and meta-analysis. PLoS ONE.

[10] Gillies R.J., Kinahan P.E., Hricak H. (2015). Radiomics: Images are more than pictures, they are data. Radiology.

[11] Bakas S., Sako C., Akbari H., Bilello M., Sotiras A., Shukla G., Rudie J.D., Santamaría N.F., Kazerooni A.F., Pati S. (2022). The University of Pennsylvania glioblastoma (UPenn-GBM) cohort: Advanced MRI, clinical, genomics, & radiomics. Sci. Data.

[12] Han L., Kamdar M.R. MRI to MGMT: Predicting methylation status in glioblastoma patients using convolutional recurrent neural networks. Proceedings of the PACIFIC SYMPOSIUM ON BIOCOMPUTING 2018: Proceedings of the Pacific Symposium.

[13] Yogananda C., Shah B., Nalawade S., Murugesan G., Yu F., Pinho M., Wagner B., Mickey B., Patel T., Fei B. (2021). MRI-based deep-learning method for determining glioma MGMT promoter methylation status. Am. J. Neuroradiol..

[14] Chen S., Xu Y., Ye M., Li Y., Sun Y., Liang J., Lu J., Wang Z., Zhu Z., Zhang X. (2022). Predicting MGMT Promoter Methylation in Diffuse Gliomas Using Deep Learning with Radiomics. J. Clin. Med..

[15] He K., Zhang X., Ren S., Sun J. Deep residual learning for image recognition. Proceedings of the IEEE Conference on Computer Vision and Pattern Recognition.

[16] Das S. (2022). Optimizing prediction of MGMT promoter methylation from MRI scans using adversarial learning. arXiv.

[17] Baid U., Ghodasara S., Mohan S., Bilello M., Calabrese E., Colak E., Farahani K., Kalpathy-Cramer J., Kitamura F.C., Pati S. (2021). The rsna-asnr-miccai brats 2021 benchmark on brain tumor segmentation and radiogenomic classification. arXiv.

[18] Saeed N., Hardan S., Abutalip K., Yaqub M. (2022). Is it Possible to Predict MGMT Promoter Methylation from Brain Tumor MRI Scans using Deep Learning Models?. arXiv.

[19] Tan M., Le Q. Efficientnet: Rethinking model scaling for convolutional neural networks. Proceedings of the International Conference on Machine Learning.

[20] Rohlfing T., Zahr N.M., Sullivan E.V., Pfefferbaum A. (2010). The SRI24 multichannel atlas of normal adult human brain structure. Hum. Brain Mapp..

[21] Isensee F., Schell M., Pflueger I., Brugnara G., Bonekamp D., Neuberger U., Wick A., Schlemmer H.P., Heiland S., Wick W. (2019). Automated brain extraction of multisequence MRI using artificial neural networks. Hum. Brain Mapp..

[22] Clark M.C., Hall L.O., Goldgof D.B., Velthuizen R., Murtagh R., Silbiger M.S. (2017). Unsupervised brain tumor segmentation using knowledge-based fuzzy techniques. Fuzzy and Neuro-Fuzzy Systems in Medicine.

[23] Fletcher-Heath L.M., Hall L.O., Goldgof D.B., Murtagh F.R. (2001). Automatic segmentation of non-enhancing brain tumors in magnetic resonance images. Artif. Intell. Med..

[24] Gupta T., Gandhi T.K., Gupta R., Panigrahi B.K. (2020). Classification of patients with tumor using MR FLAIR images. Pattern Recognit. Lett..

[25] Roozpeykar S., Azizian M., Zamani Z., Farzan M.R., Veshnavei H.A., Tavoosi N., Toghyani A., Sadeghian A., Afzali M. (2022). Contrast-enhanced weighted-T1 and FLAIR sequences in MRI of meningeal lesions. Am. J. Nucl. Med. Mol. Imaging.

[26] Ramachandram D., Taylor G.W. (2017). Deep multimodal learning: A survey on recent advances and trends. IEEE Signal Process. Mag..

[27] Chollet F. Xception: Deep learning with depthwise separable convolutions. Proceedings of the IEEE Conference on Computer Vision and Pattern Recognition.

[28] Sundararajan M., Taly A., Yan Q. Axiomatic attribution for deep networks. Proceedings of the International Conference on Machine Learning.

[29] Zeiler M.D., Fergus R. Visualizing and understanding convolutional networks. Proceedings of the European Conference on Computer Vision.

[30] Kokhlikyan N., Miglani V., Martin M., Wang E., Alsallakh B., Reynolds J., Melnikov A., Kliushkina N., Araya C., Yan S. (2020). Captum: A unified and generic model interpretability library for pytorch. arXiv.

